# Comparison of the adjuvant effect of conbercept intravitreal injection at different times before vitrectomy for proliferative diabetic retinopathy

**DOI:** 10.3389/fendo.2023.1171628

**Published:** 2023-05-26

**Authors:** Zhikun Yang, Yu Di, Junjie Ye, Weihong Yu, Zijian Guo

**Affiliations:** ^1^ Department of Ophthalmology, Peking Union Medical College Hospital, Chinese Academy of Medical Sciences, Beijing, China; ^2^ Key Laboratory of Ocular Fundus Disease, Chinese Academy of Medical Sciences and Peking Union Medical College, Beijing, China; ^3^ Department of Clinical Laboratory, Peking Union Medical College Hospital, Chinese Academy of Medical Sciences, Beijing, China

**Keywords:** conbercept, proliferative diabetic retinopathy, pars plana vitrectomy, vascular endothelial growth factor, adjuvant effect

## Abstract

**Purpose:**

To assess the optimal time of intravitreal conbercept (IVC) treatment prior to pars plana vitrectomy (PPV) in patients with severe proliferative diabetic retinopathy (PDR).

**Method:**

This study was exploratory in nature. Forty-eight consecutive patients (48 eyes) with PDR were divided into four groups according to different IVC times (0.5 mg/0.05 mL) before PPV: group A (3 days), group B (7 days), group C (14 days), and group D (non-IVC). Intraoperative and postoperative effectiveness were assessed, and vitreous VEGF concentrations were detected.

**Result:**

For intraoperative effectiveness, groups A and D had a higher incidence of intraoperative bleeding than groups B and C (*P* = 0.041). Furthermore, groups A-C required less surgical time than group D (*P* < 0.05). For postoperative effectiveness, group B had a significantly higher proportion of visual acuity that improved or remained unchanged than group D (*P* = 0.014), and groups A-C had lower proportions of postoperative bleeding than group D. The vitreous VEGF concentration of group B (67.04 ± 47.24 pg/mL) was significantly lower than that of group D (178.29 ± 110.50 pg/mL) (*P* = 0.005).

**Conclusion:**

IVC treatment that was administered 7 days preoperatively was associated with better effectiveness and a lower vitreous VEGF concentration than its administration at other time points.

## 1 Introduction

Diabetes mellitus (DM) is a chronic, noncommunicable, multisystem disease that has reached epidemic proportions. By 2040, the proportion of the world’s adult population with DM is expected to increase to 10.4%, translating to 642 million patients with diabetes (1). Diabetic retinopathy (DR) is an ocular microvascular complication of DM, and it is the most common cause of blindness worldwide (2). The severe stage of DR is proliferative diabetic retinopathy (PDR), defining characteristic of which is neovascularization, and tractional retinal detachment (TRD) are common complications (3). Pars plana vitrectomy (PPV) is an established management method for advanced complications of PDR (4–6). However, intraoperative bleeding generally occurs during epiretinal neovascular membrane peeling, which may necessitate more surgical maneuvers, increase instrument replacement frequency, and lengthen surgical time. Therefore, many researchers have used anti-vascular endothelial growth factor (VEGF) as an adjuvant treatment before PPV in patients with severe PDR and found that it can reduce the incidence of intraoperative bleeding and the total surgical time (7–10)

Conbercept (KH902; Chengdu Kanghong Biotech Co., Sichuan, China) is a novel recombinant fusion protein that can specifically bind to VEGF-A, VEGF-B, and placental growth factor (PLGF) (11). It has been widely used in treating retinal vascular diseases, such as neovascular age-related degeneration, diabetic macular edema, and retinal vein occlusion (12–14). The effect of conbercept on vitrectomy for PDR was initially reported in 2016, when it was proposed that intravitreal conbercept injection (IVC) before PPV could minimize the risk of intraoperative bleeding (15). In most studies, the researchers have performed IVC 3 to 14 days before PPV in patients with PDR, but the optimal time-point for IVC remains controversial (8, 16–19). This lack of clarity may decrease the effect of IVC application in some cases. Furthermore, most studies have evaluated intraoperative and postoperative effectiveness without investigating the mechanism at the molecular level.

The present study was performed to compare the adjuvant effect of IVC at different times before vitrectomy for PDR and to determine the optimal time for injection. Furthermore, we investigated the changes in VEGF concentration in the vitreous humor of patients with severe PDR after IVC to better understand the mechanisms at the molecular level.

## 2 Methods

### 2.1 Patients

This study was exploratory in nature. Forty-eight consecutive patients (48 eyes) with PDR were diagnosed at the Peking Union Medical College Hospital (PUMCH) Department of Ophthalmology and assigned to four groups by sortation randomization method. Data were collected between February 2016 and April 2022. The study was approved by the hospital’s institutional review board (NO. HS-1035) and complied with the Declaration of Helsinki. The inclusion criteria were (1) aged 18 years or older; (2) diagnosed with type 2 DM; (3) diagnosed with severe PDR (presence of VH with proliferation or TRD) and requiring surgical intervention; and (4) pan-retinal photocoagulation (PRP) was not applicable or could not be completed because of non-absorbable VH or retinal detachment by neovascularized fibrovascular membrane. The exclusion criteria were (1) received an intravitreal injection of any anti-VEGF within 3 months; (2) received an intravitreal dexamethasone implant within 6 months or an intravitreal injection of steroids within 3 months; (3) a history of vitrectomy; (4) existence of diseases other than DR, e.g., retinal vein occlusion and choroidal neovascularization; (5) existence of eye infection and intraocular inflammation; or (6) existence of severe systemic diseases, such as uncontrolled diabetes, uncontrolled hypertension, recent myocardial infarction or cerebral vascular accident.

### 2.2 Ocular examinations and laboratory assessment

Before IVC and after PPV, all patients received comprehensive ophthalmic examinations, including best-corrected visual acuity (BCVA) measurement, intraocular pressure (IOP) evaluation, anterior segment slit-lamp examination, and fundus examination. In addition, axial length, corneal curvature, and corneal endothelial cell count were assessed before PPV. B-scan ultrasonography was used to observe the degree of VH, vitreous proliferation, and retinal detachment. Fasting blood glucose (FBG) and 2-h postprandial blood glucose (2 h-PBG) data were also collected at the time of enrollment.

### 2.3 Treatment procedures

Patients were divided into four groups according to the time of IVC (0.5 mg/0.05 mL) before PPV: group A (3 days), group B (7 days), group C (14 days), and group D (non-IVC). All the patients signed an informed consent form prior to each IVC and PPV.

For IVC, all patients received levofloxacin eye drops four times a day for 3 days before the injection. Following topical anesthesia and sterilization of the operating field, a 30-gauge needle was inserted into the superior temporal pars plana (4 mm posterior to the limbus), and 0.5 mg (0.05 mL) of conbercept was injected. Then, ofloxacin eye ointment was administered after the IOP returned to normal. All patients were followed up 1 day after IVC, and levofloxacin eye drops were applied for another 3 days.

For PPV, all surgeries were performed by the same experienced retina surgeon (Junjie Ye) using a 23G vitrectomy system. After vitrectomy, posterior vitreous detachment, fibrovascular membrane delamination, and retinal photocoagulation were performed as needed. Intraoperative hemostasis was obtained by increasing the perfusion pressure, fluid/air exchange, or endodiathermy. Silicone oil or perfluoropropane (C3F8) tamponade was employed when necessary.

To ensure the comparability of all the indicators between the groups, we used the “VH grading system” to assess the severity of VH after IVC and the “complexity score” to evaluate the difficulty of PPV. The grading criteria for VH were as follows: Grade 0, no VH; Grade 1, mild VH with visible fundus details, but with difficulty observing the retina nerve fiber layer or small vessels; Grade 2, moderate VH with visible optic disc and large vessels; Grade 3, severe VH with faint fundus reflex, only optic disc visible; Grade 4, very severe VH with no fundus reflex and no view of the fundus (20). The complexity score was graded by quantifying (1) the fibrovascular proliferation number (each quadrant involved corresponds to one point); (2) the fibrovascular proliferation location: posterior to the equator (zero points), anterior to the equator (one point), both anterior and posterior (two points); (3) TRD (one point), tractional rhegmatogenous retinal detachment (two points); and (4) the absence of posterior vitreous detachment (one point) (21).

### 2.4 Vitreous humor collection and testing

We collected 1.0 mL of undiluted vitreous humor at the beginning of surgeries (cataract or PPV) into a 1.5 mL sterile Eppendorf tube; then, it was frozen at −80°C until testing. The vitreous humor samples were thawed at room temperature, and VEGF was measured by an enzyme-linked immunosorbent assay kit (R&D Systems, Minneapolis, MN, USA) according to the manufacturer’s protocol.

### 2.5 Intraoperative and postoperative effectiveness assessment

Intraoperative effectiveness was assessed using the following parameters: intraoperative bleeding (macroscopic bleeding in the surgery especially during the process of fibrotic membrane dissection), hemostatic techniques (increasing the perfusion pressure, fluid/air exchange, or endodiathermy), intraocular tamponade (silicone oil, C3F8, or intraocular irrigation solution), and total surgical time (the time from the first incision to the time of final surgical closure). Additionally, BCVA, IOP, anterior inflammation, and postoperative bleeding (including vitreous hemorrhage, retinal hemorrhage, and early postoperative hyphema) at 3 days after PPV were used to assess early postoperative effectiveness.

### 2.6 Statistical analysis

Statistical analyses were conducted using SPSS statistical software version 26.0 (IBM, Armonk, NY, USA). Continuous variables are presented as the mean ± standard deviation (SD) or median (quartile range, QR), and categorical variables are presented as numbers and percentages. A Kolmogorov-Smirnova test was used to test the parameter distribution. For continuous variables, the one-way analysis of variance or the Kruskal-Wallis test was used to compare parameters between different groups, and the paired t-test or nonparametric Wilcoxon signed-rank test was used to compare parameters between the initial (before IVC) and final (3 days after PPV) visits. In addition, Pearson’s chi-squared test or Fisher’s exact test was performed to compare categorical variables. The differences in the data are reported with 95% confidence intervals (CIs). A two-tailed P value of ≤0.05 was considered statistically significant for all analyses.

## 3 Results

### 3.1 Demographics and clinical characteristics

Forty-eight patients (48 eyes) with type 2 DM were included in the study, of which 25 were male and 23 were female, and their mean age was 51.5 ± 9.8 years (range, 27-69 years). The mean duration of DM was 12.84 ± 6.58 years (range, 0.3-26 years). At the time of enrollment, the FBG and 2 h-PBG were 7.12 ± 2.16 mmol/L and 9.40 ± 2.76 mmol/L, respectively.

Among the 48 eyes with severe PDR, there were 26 right eyes and 22 left eyes. The median value of BCVA was 1.85 LogMAR, and the mean value of IOP was 14.02 mmHg. The severity of VH after IVC was as follows: Grade 0, two eyes; Grade 1, thirteen eyes; Grade 2, five eyes; Grade 3, eleven eyes; and Grade 4, seventeen eyes. The PPV complexity score was 5.21 ± 2.43. One eye, 1 eye, 4 eyes, and 5 eyes underwent lensectomy in groups A, B, C, and D, respectively.

There were 12 patients (12 eyes) in each group (groups A-D). We found no statistically significant differences in age, duration of DM, FBG, 2 h-PBG, BCVA, IOP, VH grade, or PPV complexity score among the subgroups (**Table 1**).

**Table 1:** Demographic characteristics and initial ocular manifestations of patients with proliferative diabetic retinopathy.

	Group A	Group B	Group C	Group D	F/H	P -value
Ages (years)	44.50 ± 11.77	51.17 ± 10.44	54.92 ± 6.82	55.33 ± 5.65	3.704#	0.018
Gender						
Male	2 (16.67%)	3 (25%)	7 (58.33%)	5 (41.67%)	5.132	0.184
Female	10 (83.33%)	9 (75%)	5 (41.67%)	7 (58.33%)		
Duration of DM	11.94 ± 7.35	9.50 ± 4.62	14.67 ± 7.62	15.25 ± 5.33	2.087#	0.116
FBG (mmol/L)	5.85 (2.38)	6.65 (4.15)	7.70 (3.10)	6.90 (4.07)	3.669	0.300
2h-PBG (mmol/L)	8.86 ± 2.95	10.03 ± 2.64	10.57 ± 3.22	8.13 ± 1.60	2.050#	0.121
BCVA (LogMAR)	1.85 (1.28)	2.08 (0.45)	1.85 (0.56)	1.15 (1.0)	5.702	0.127
IOP (mmHg)	14.83 ± 3.51	13.67 ± 2.96	14.08 ± 3.37	13.51 ± 2.88	1.360#	0.715
Grading of VH						
Grade 0	0 (0.00%)	1 (8.33%)	1 (8.33%)	0 (0.00%)	2.197	1.000
Grade 1	4 (33.33%)	4 (33.33%)	1 (8.33%)	4 (33.33%)	3.058	0.406
Grade 2	2 (16.67%)	0 (0.00%)	2 (16.67%)	1 (8.33%)	2.512	0.734
Grade 3	3 (25.00%)	2 (16.67%)	3 (25.00%)	3 (25.00%)	0.560	1.000
Grade 4	3 (25.00%)	5 (41.67%)	5 (41.67%)	4 (33.33%)	1.102	0.909
Complexity score of PPV	5.17 ± 2.29	4.92 ± 2.50	5.50 ± 2.61	5.25 ± 2.60	0.111#	0.953

DM, diabetes mellitus; FBG, fasting blood-glucose; PBG, posprandial blood glucose; VH, vitreous. hemorrhage; PPV, pars plana vitrectomy. # means F value: one-way analysis of variance test, *P-value < 0.05.

### 3.2 Intraoperative effectiveness assessment

There was a statistically significant difference in the proportion of patients who experienced intraoperative bleeding among the subgroups (P = 0.039) (**Table 2**). Group A (nine eyes, 75%) and group D (nine eyes, 75%) had a higher proportion of patients with intraoperative bleeding than group B (four eyes, 33.33%) and group C (three eyes, 33.33%) (P = 0.041). There was no statistically significant difference in the utilization of different hemostatic techniques among the subgroups (P = 0.095, 0.600, 0.737) (**Table 2**). There was a statistically significant difference in the proportion of patients who achieved spontaneous hemostasis among the subgroups (P = 0.016) (**Table 2**), and more patients in group A (six eyes, 50%) achieved spontaneous hemostasis than in group D (0 eyes, 0%) (P = 0.014) (**Table 3**).

**Table 2:** Intraoperative and postoperative effectiveness assessment of preoperative IVC treatment of patients with proliferative diabetic retinopathy.

	Group A	Group B	Group C	Group D	F/H	P -value
Intraoperative bleeding	9 (75%)	4 (33.33%)	4 (33.33%)	9 (75%)	8.392	0.039*
Hemostatic techniques						
Increasing the infusion bottle height	0 (0.00%)	2 (16.67%)	2 (16.67%)	5 (41.67%)	6.436	0.095
Fluid/air exchange	1 (8.33%)	0 (0.00%)	0 (0.00%)	2 (16.67%)	3.097	0.600
Endodiathermy	2 (16.67%)	0 (0.00%)	1 (8.33%)	2 (16.67%)	2.290	0.737
Spontaneous. hemostasis	6 (50.00%)	2 (16.67%)	1 (8.33%)	0 (0.00%)	9.537	0.016*
Intraocular tamponade						
Silicon oil	8 (66.67%)	8 (66.67%)	8 (66.67%)	9 (75%)	7.024	0.057
C3F8	3 (25.00%)	0 (0.00%)	0 (0.00%)	2 (16.67%)	6.932	0.200
Intraocular irrigation	1 (8.33%)	4 (33.33%)	2 (16.67%)	3 (25.00%)	7.415	1.000
Total surgical time (minutes)	67.5 ± 29.43	51.83 ± 12.63	55.75 ± 20.00	87.33 ± 21.13	6.518# F	0.001*
BCVA						
Improved or remained unchanged	9 (75.00%)	12 (100.00%)	10 (83.33%)	6(50.00%)	8.845	0.031*
Decreased	3 (25.00%)	0 (0.00%)	2 (16.67%)	6 (50.00%)		
IOP (mmHg)	15.25 ± 2.93	14.92 ± 4.29	16.00 ± 5.94	15.17 ± 2.86	0.148#	0.930
Intraocular inflammation	6 (50.00%)	5 (41.67%)	5 (41.67%)	5 (41.67%)	1.797	0.701
Postoperative bleeding	5 (41.67%)	5 (41.67%)	6 (50.00%)	8 (66.67%)	2.000	0.705

BCVA, best corrected visual acuity. ^#^means F value: one-way analysis of variance test, *P-value < 0.05.

**Table 3:** The differences between groups in intraoperative bleeding and spontaneous hemostasis.

Intraoperative bleeding				Spontaneous hemostasis		
	Group A	Group B	Group C	Group A	Group B	Group C
Group B	0.041*			0.193		
Group C	0.041*	1.000		0.069	1.000	
Group D	1.000	0.041*	0.041*	0.014*	0.478	1.000

*P-value < 0.05.

There was a statistically significant difference in the total surgical time among the subgroups (P = 0.001) (**Table 2**), and group D (87.33 ± 21.13 min) had a statistical longer total surgical time than groups B (51.83 ± 12.63 min), and C (55.75 ± 20.00 min) (P = 0.001, 0.005). However, there was no statistically significant difference between groups A, B, and C (P > 0.05) (**Table 4**).

**Table 4:** The differences between groups in total surgical time and improved or remained unchanged BCVA.

Total surgical time				Improved or remained unchanged BCVA		
	Group A	Group B	Group C	Group A	Group B	Group C
Group B	0.299			0.217		
Group C	0.549	0.971		1.000	0.478	
Group D	0.127	0.001*	0.005*	0.400	0.014*	0.193

*P-value < 0.05.

### 3.3 Early postoperative effectiveness assessment

Compared with the initial BCVAs in the patients, the proportion of patients with a visual acuity that improved or remained unchanged in group B was significantly higher than those in group D (*P* = 0.014) (**Table 4**). There was no statistically significant difference in IOP between the subgroups (*P* = 0.930). Only one eye had an elevated IOP (29 mmHg) in group C, which could be controlled by hypotensive eye drops. Although there was no statistically significant difference in the proportion of patients with postoperative bleeding among the subgroups (*P* = 0.705), groups A, B, and C had lower proportions than group D (**Table 2**).

### 3.4 VEGF concentration in vitreous humor

**Figure 1:**
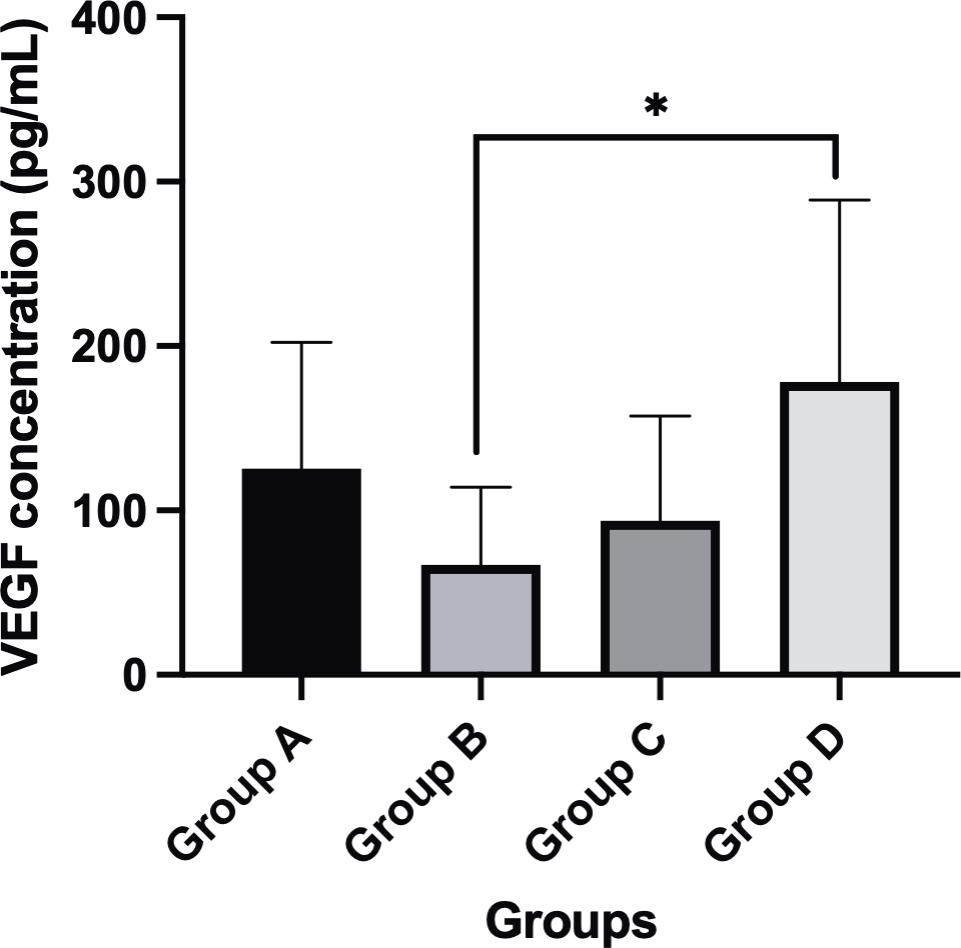
Comparison of VEGF concentrations (mean value) in different subgroups after IVC treatment. *P-value < 0.05.

The mean VEGF concentrations in the vitreous humor were 125.34 ± 76.89 pg/mL, 67.04 ± 47.24 pg/mL, 93.81 ± 63.57 pg/mL, and 178.29 ± 110.50 pg/mL in groups A-D, respectively. There was a statistically significant difference among the subgroups (*P* = 0.032). The VEGF concentrations in the vitreous humor in group B were significantly lower than those in group D (*P* = 0.005). However, there were no significant differences between groups A, C, and D (*P >* 0.05) (**Figure 1**).

### 3.5 Adverse events

No ocular or systemic adverse events were observed after IVC and PPV.

## 4 Discussion

In this study, we compared the adjuvant effect of IVC at different times before vitrectomy for PDR and investigated the vitreous VEGF concentrations in the patients with severe PDR. In terms of intraoperative effectiveness, we found that group with IVC 3 days prior to vitrectomy and group with non-IVC had a higher incidence of intraoperative bleeding than groups with IVC 7 days and 14 days preoperatively. However, group with IVC 3 days prior to vitrectomy had a higher proportion of patients who achieved spontaneous hemostasis than group with non-IVC. Furthermore, groups with 3 days, 7 days, and 14 days prior to vitrectomy required less total surgical time than group with non-IVC. In terms of postoperative effectiveness, group with 3 days prior to vitrectomy had a significantly higher proportion of patients with a visual acuity that improved or remained unchanged than group with non-IVC, and groups with 3 days, 7 days, and 14 days prior to vitrectomy had lower a proportion of patients who experienced postoperative bleeding than group with non-IVC. Group with 3 days prior to vitrectomy had a significantly lower concentration vitreous VEGF concentration (67.04 ± 47.24 pg/mL) than group with non-IVC (178.29 ± 110.50 pg/mL).

Anti-VEGF treatment has been used preoperatively to reduce vascular proliferation and the vascularity of neovascular tissue. Several studies have investigated the efficiency of anti-VEGF treatment before PPV in patients with PDR (7–9, 15, 19). Hattori et al. (22) found better adjunct effect on PDR patients with intravitreal bevacizumab (IVB) at 3 days before PPV than non- IVB before PPV. Furthermore, Castillo et al. (7) performed a study to assess the optimal interval of intravitreal bevacizumab (IVB) administration in diabetic patients undergoing PPV for severe PDR. They found better postoperative outcomes at 6 months when subjects received preoperative IVB at 5 to 10 days before PPV compared to 1-3 days for the treatment of PDR-related complications. In addition, Wang et al. (23) performed a meta-analysis to compare the efficiency of different perioperative time points of anti-VEGF treatment in patients who underwent PPV for severe PDR. They reported that anti-VEGF treatment at 6 to 14 days before PPV could significantly improve post-operative BCVA, decrease the incidence of recurrent vitreous hemorrhage, as well as reduce the duration of surgery. In our study, we found the administration of IVC treatment at 7 days preoperatively was more effective than the administration of the treatment at other time points in reducing the incidence of intraoperative and postoperative bleeding, shortening the surgical time, improving early postoperative BCVA, and decreasing vitreous VEGF concentration.

In terms of intraoperative bleeding, Simunovic et al. (24) performed a meta-analysis of 22 randomized control trials and reported that the use of anti-VEGF treatment before PPV results in less intraoperative bleeding. Furthermore, Wang et al. (23) reported that anti-VEGF treatment at 1 to 5 days, 6 to 14 days, and more than 14 days before PPV significantly reduced the incidence of intraoperative bleeding. In our study, we found that the incidence of intraoperative bleeding after receiving IVC at 7 days and 14 days was lower than that of receiving IVC at 3 days and in the control group. The results might be related to the time conbercept reached the peak concentration. Li et al. (25) investigated the ocular pharmacokinetics of rabbits following a single dose of IVC (0.5 mg) and found that the vitreous half-life of conbercept was 4.24 days. Moreover, Du et al. (26) observed total VEGF levels in hyperglycemic mouse eyes and reported that the conbercept concentration remained at high levels for 4 days. Thus, we speculated that preoperative IVC treatment at 3 days may not provide enough time for neovascular regression. Although Mao et al. (27) proposed that administering IVC 3 to 7 days before PPV to manage PDR could reduce the incidence of intraoperative bleeding, and the mean time (4.95 days) of IVC administration before PPV was longer than the half-life (4.24 days) of conbercept.

The reduction in surgical time after preoperative IVC was similar to that after other anti-VEGF agents. Li et al. (16) performed a prospective randomized study to assess the clinical efficiency of preoperative IVC treatment for severe PDR patients. The results demonstrated that IVC treatment at 7 days and 14 days significantly reduced the total surgery time when compared to the control group. Furthermore, Gao et al. (19) found that preoperative IVC treatment at 3 to 5 days also reduced the duration of surgery. Likewise, we found that the patients who received IVC treatment at 3 days, 7 days, and 14 days had shorter total surgery times than the control group. The surgical time reduction was mainly because anti-VEGF agents can cause fibrovascular proliferative membrane retraction and shrinkage, which makes manipulative techniques and visualization easier during surgery. In addition, we were able to manage most membrane dissections in IVC patients without the use of endodiathermy or blood aspiration, thereby reducing the need for tool exchange and shortening the surgical time.

Wakabayashi et al. (28) proposed that high vitreous levels of VEGF prior to vitrectomy are associated with high neovascular activity and may cause extensive proliferative changes. Therefore, such patients may be more susceptible to postoperative bleeding after PPV. Conbercept is a fully humanized, soluble, VEGF receptor protein. The most notable characteristic of conbercept is that it binds not only to VEGF-A but also to VEGF-B and PIGF (11). Some studies have reported that preoperative IVC treatment caused less postoperative bleeding (10, 19, 26). In our study, we found that the VEGF concentrations of groups A-C (125.34 ± 76.89 pg/mL, 67.04 ± 47.24 pg/mL, 93.81 ± 63.57 pg/mL, respectively) were lower than those of group D (178.29 ± 110.50 pg/mL), and the incidence of postoperative bleeding in groups A-C was lower than that of group D. Thus, we speculated that postoperative bleeding might be related to the vitreous VEGF concentration. Furthermore, Nistic et al. (29) suggested that intraoperative bleeding increases the risk of postoperative bleeding. In our study, the incidence of intraoperative bleeding was not significantly different between group A and group D, but group A had a higher proportion of patients who achieved spontaneous hemostasis than group D. Therefore, this could explain why group A had less patients with postoperative bleeding than group D.

Numerous factors might be associated with postoperative BCVA, such as a history of TRD, surgical trauma, postoperative bleeding, and silicone oil tamponade. Chen et al. (30) performed a meta-analysis and proposed that preoperative IVC achieved better BCVA outcomes. Furthermore, Castillo et al. (7) reported better postoperative BCVA at 6 months when subjects received preoperative anti-VEGF 5-10 days before PPV for PDR as opposed to 1-3 days. Wang et al. (23) also found that preoperative anti-VEGF treatment at 6 to 14 days significantly improved postoperative BCVA when compared with that of the sham group. Similarly, we found that group B had a significantly higher proportion of patients with a visual acuity that improved or remained unchanged than group D. This might be related to significantly less intraoperative and postoperative bleeding, shorter total surgical times, and fewer intraoperative manipulations in group B than in group D.

Zhang et al. (31) reported that concentrations of VEGF-A and VEGF-B decreased significantly in the vitreous humor of patients with PDR after IVC. Furthermore, Hu et al. (17) detected intraocular proangiogenic profibrotic cytokine profiles within 7 days after IVC in patients with PDR. They reported that the vitreous VEGF-A concentration decreased ten-fold at day 2 and remained at a low level at 3 days, 4 days, 5 days, 6 days, and 7 days. Nevertheless, our results showed that the vitreous VEGF concentration in group B (7 days, 67.04 ± 47.24 pg/mL) was lower than that in group A (3 days, 125.34 ± 76.89 pg/mL) and in group C (14 days, 93.81 ± 63.57 pg/mL). This might be attributed to the fact that our study detected all VEGF family members, including not only VEGF-A and VEGF-B but also VEGF-C and VEGF-D. VEGF-C and VEGF-D also play a role in ocular angiogenesis. Hu et al. (17) identified that the expression levels of VEGF-C and VEGF-D were upregulated with the remarkable inhibition of VEGF-A by IVC. VEGF-A, VEGF-C, and VEGF-D share a common receptor, namely, VEGFR2; thus, with the downregulation of VEGF-A by anti-VEGF drugs, VEGF-C and VEGF-D might be upregulated in a feedback manner. In the study by Hu et al. (17), they found that the total concentrations of VEGF-A, VEGF-B, and VEGF-C at 7 days were also lower than those at 2 days, 3 days, 4 days, 5 days, and 6 days.

The present study has several limitations. First, the small sample size is the major limitation and may lead to an overestimation of the preoperative IVC effect in our study. Additional multicenter studies with larger samples may be needed. Second, the follow-up time was short in our study. Yang et al. (15) proposed that preoperative IVC may reduce the incidence of early postoperative bleeding in patients undergoing diabetic vitrectomy. However, it did not significantly reduce the incidence of late postoperative bleeding. Therefore, we only performed short-term follow-ups on patients to evaluate early postoperative effects. Third, Hu et al. (17) found that the vitreous VEGF-A concentration decreased ten-fold at day 2 and remained at a low level at 3 days, 4 days, 5 days, 6 days, and 7 days. Thus, we should measure the VEGF concentration at 1 day or two days after IVC. Finally, we detected only the VEGF concentration in this study. Zhang et al. (30) proposed that PIGF may be an attractive molecular target in the prevention of pathological angiogenesis in PDR. Furthermore, the PIGF concentration must be detected to investigate the preoperative IVC effect in PDR patients at a deeper molecular level.

In conclusion, our study revealed that IVC treatment was a potentially effective adjunctive therapy prior to PPV for severe PDR. Additionally, the administration of IVC treatment at 7 days preoperatively was more effective than the administration of the treatment at other time points in reducing the incidence of intraoperative and postoperative bleeding, shortening the surgical time, improving early postoperative BCVA, and decreasing vitreous VEGF concentration.

## Data availability statement

The raw data supporting the conclusions of this article will be made available by the authors, without undue reservation.

## Ethics statement

The studies involving human participants were reviewed and approved by the Ethics Committee of Peking Union Medical Hospital (NO. HS-1035). The patients/participants provided their written informed consent to participate in this study.

## Author contributions

(I)Conception and design: JY. (II) Provision of study materials or patients: JY and ZY. (III) Collection and assembly of data: YD, JY, WY, and ZG; Data analysis and interpretation: YD and. ZY. All authors contributed to the article and approved the submitted version.
